# Towards the Design and Implementation of an Image-Based Navigation System of an Autonomous Underwater Vehicle Combining a Color Recognition Technique and a Fuzzy Logic Controller

**DOI:** 10.3390/s21124053

**Published:** 2021-06-12

**Authors:** Yu-Hsien Lin, Chao-Ming Yu, Chia-Yu Wu

**Affiliations:** Department of Systems & Naval Mechatronic Engineering, National Cheng-Kung University, Tainan City 70101, Taiwan; vicfish@gmail.com (C.-M.Y.); karol117203@gmail.com (C.-Y.W.)

**Keywords:** AUV, image navigation system, fuzzy logic controller, object tracking

## Abstract

This study proposes the development of an underwater object-tracking control system through an image-processing technique. It is used for the close-range recognition and dynamic tracking of autonomous underwater vehicles (AUVs) with an auxiliary light source for image processing. The image-processing technique includes color space conversion, target and background separation with binarization, noise removal with image filters, and image morphology. The image-recognition results become more complete through the aforementioned process. After the image information is obtained for the underwater object, the image area and coordinates are further adopted as the input values of the fuzzy logic controller (FLC) to calculate the rudder angle of the servomotor, and the propeller revolution speed is defined using the image information. The aforementioned experiments were all conducted in a stability water tank. Subsequently, the FLC was combined with an extended Kalman filter (EKF) for further dynamic experiments in a towing tank. Specifically, the EKF predicts new coordinates according to the original coordinates of an object to resolve data insufficiency. Consequently, several tests with moving speeds from 0.2 m/s to 0.8 m/s were analyzed to observe the changes in the rudder angles and the sensitivity of the propeller revolution speed.

## 1. Introduction

With the mature development of marine engineering, underwater technology has gradually become significant. The University of Washington constructed the first self-propelled underwater research vehicle (SPURV) for the research of underwater diffusion effects and acoustic transmission [1]. Underwater vehicles have gradually become autonomous, and more precise missions are possible with computers and electronic technologies. Allen, et al. [2] developed a new generation AUV through the reduction of cost and volume, and these have been successfully applied in scientific research and military contexts. Due to the influence of the unknown and uncertain factors of pressure, light, and ocean currents in the deep-sea environment, underwater technicians encounter safety threats and other difficulties in conducting missions.

In deep water where human missions are not possible, AUVs equipped with machine vision processing technology can be applied. Machine vision processing technology mainly employs cameras and computers to analyze images and provide control information of the AUV drive system. There have been numerous research studies on the application of an AUV equipped with machine vision processing technology, such as the detection of underwater man-made structures and pipeline detection [3,4,5,6,7,8,9,10], auxiliary sonar image navigation [11,12], simultaneous localization and mapping (SLAM) [13,14,15,16,17], obstacle avoidance [18,19], identifying and tracking the habitats of sea animals [20], underwater docking systems [21,22,23,24,25,26,27], and object tracking [28,29,30,31,32,33,34,35,36].

Thereupon, AUV navigation has become a major challenge in the research field. Particularly, path planning and object tracking are critical for the successful operation of AUVs. There have been various research studies discussing AUV navigation technologies [37,38,39,40,41,42]. Specifically, optical guidance is conducted through the visual system consisting of charge-coupled device (CCD) cameras and frame grabbers [21]. In addition, the processed images contain much information on the environment [43]. Compared with other underwater sensors, optical devices are generally considered to be less expensive but provide abundant information. Bazeille, et al. [44] identified underwater objects through the detection of all image colors known to be compatible. Yu, et al. [45] demonstrated that color extraction could be used for the detection of artificial underwater boundary markers and studied multiple colors that can be observed in underwater environments.

The purpose of this study is to develop an AUV underwater image-based navigation system with a combination of color recognition and fuzzy logic controllers (FLCs). In the experiment, a red ball is considered as an underwater object, and a dual-lens camera is used to capture images for processing. Since environmental factors (such as turbidity and brightness) can cause errors in image recognition, binarization can be used to separate objects from backgrounds. The contours can then be completed through noise removal and morphology. The image information obtained provides the input values of the FLC, which are used to control servo motors and propellers in this study. Eventually, an extended Kalman filter (EKF) would be integrated into the control system and be used to realize the data estimation of the dynamic guidance experiment.

For the remainder of this paper, Section 2 illustrates the AUV exterior design. Section 3 introduces the recognition of the image-processing method used in this study. In Section 4, the image-based navigation system is discussed, and the design and parameter adjustment methods for the FLC are explained. Section 5 verifies the AUV image-based navigation system through experimental results and analysis. Section 6 provides conclusions and discussion regarding the aforementioned results.

## 2. AUV Design and Structure

The exterior design of the AUV used in this study is a torpedo type with a length of 1.8 m and a diameter of 0.17 m. The system architecture of the AUV is described in Section 2.1, Section 2.2 introduces the image-processing module as the main sensing input source, and Section 2.3 describes the function of the motion control module. 

### 2.1. System Structure of the AUV

**Figure 1:**
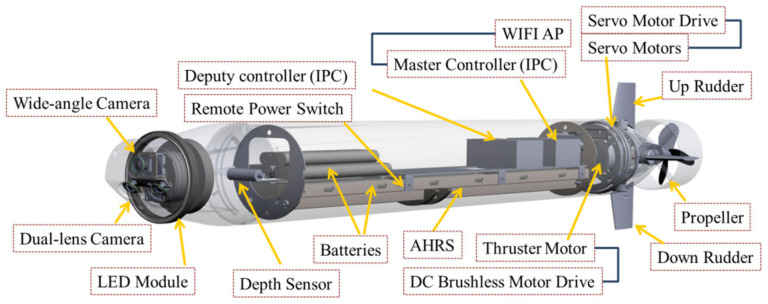
Interior structure of the AUV.

**Figure 2:**
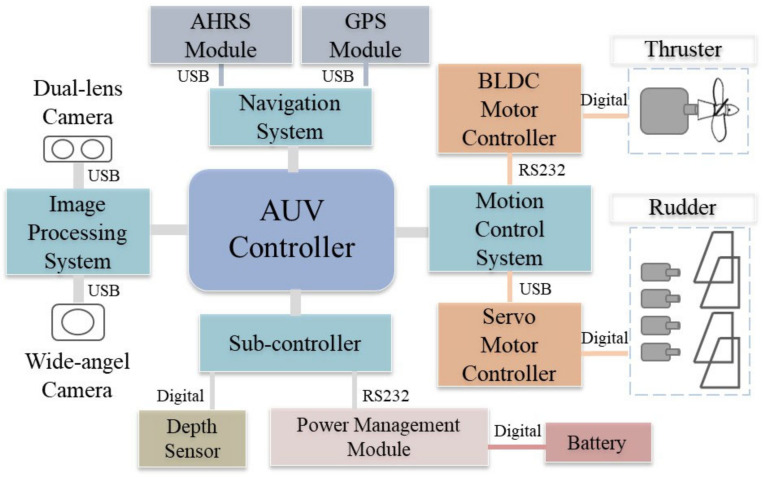
The system architecture of the AUV.

The interior structure of the AUV, such as the drive mechanism and various control components, comprises three parts: the bow, the parallel middle body, and the stern. The bow is installed with the image system and LED apertures. The parallel middle body contains the attitude and heading reference system (AHRS), electric power equipment, communication equipment, and controllers. The stern section has a propeller and four sets of independent rudders. The AHRS module used in this study includes a multi-axis magnetometer, gyroscope, and accelerometer. It can perceive the AUV’s pitch, roll, and yaw motion variables in space; an ultra-slim and fanless PC is used as the main controller for the integration of all systems, as shown in Figure 1. On the other hand, the AUV architecture is presented in Figure 2. The aim of this study is to integrate these image-processing methods into the AUV for realizing dynamic and real-time visual tracking. Since the identification and control system considered in the AUV is subject to the power consumption and the performance of the processor, it would be more efficient to use the present methods than complex deep-learning methods.

### 2.2. Image-Processing Module

In this study, the dual-lens camera installed at the front of the bow is adopted to frontal images acquisition. For close-range detection, the image information is captured by this device effectively. Related image information can be transmitted back to the main control system for processing, and object tracking can be conducted through the operation and power system. A dual-lens camera is used in the image-processing module (resolution is 1280 × 780). Particularly, the camera is suitable for particular conditions (when additional light sources are difficult to use or light sources are insufficient), featuring a low-illuminance lens. Thus, it makes the AUV have visual functions and can carry out visualization tasks. Insufficient light in underwater environments influences the precision of the visual recognition. For this purpose, an auxiliary LED light source is installed outside the bow in this study. The LED light has directionality and can increase centrality. In addition, brightness can be adjusted for comparing the recognition efficiency in various lighting conditions. 

### 2.3. Motion Control Module

Due to the influences of the AUV’s weight and resistance, the motor torque and propeller revolution speed required consideration. However, a motor with a high propeller revolution speed and low torque may fail to propel the vehicle due to excessive resistance. Therefore, a motor with a high torque and low propeller revolution speed is selected to ensure the vehicle’s ability to move underwater. In the AUV, a brushless DC (BLDC) motor is selected with an encoder, and four sets of independent servomotors are selected for the driving force of the AUV motion. The encoder plays a crucial role in the motion control module. It can track the revolution motion in real time and obtain accurate feedback data. The output signals are directly converged into digital signals to angular position and sent back to the controller via an RS232 cable. 

## 3. Recognition of Image Features

Images captured with cameras are 3D images in RGB color space; R stands for red, G for green, and B for blue. Each color has a value range of positive integers from 0 to 255, forming the color of each pixel in the image. The RGB color space is an additive color system [46], where the RGB correlation in the pixel can be too high and the difference value of the color changes caused by brightness is more challenging to express. Typically, images captured by photosensitive components, such as cameras, are easily affected by ambient brightness changes, resulting in calculation errors. Notably, light in underwater environments is subject to strong changes and effects. The HSV color space is selected for this study to reduce the effects caused by changes in ambient light sources and to increase the resolution. The HSV color space can be converted from the RGB color space as shown in Equations (1)–(3). As explained by Smith in 1978 [47], H in the HSV color space stands for hue, with the 360 degrees of the circle in the cylindrical coordinates as the value range, and all colors are arranged on this circle—for example, yellow is approximately 60°; S denotes saturation and has a range of 0–100%—smaller values indicate lower saturation and that the displayed color is close to white; and V refers to value and has a range of 0–100%, indicating the brightness displayed by the color—the smaller the value is, the darker the color is. This color space is unique in that the three visual vectors—hue, saturation, and value—are highly independent. Moreover, the differences caused by changes in the ambient light source can be defined and distinguished. Therefore, this color space is especially suitable for situations that require the independent identification of hue, saturation, and value.

(1) $$H=\left\{\begin{array}{c} 0{}^{\circ},\;\;\;if\;\left(R,G,B\right)max=\left(R,G,B\right)min\\ 60{}^{\circ}\cdot \frac{G-B}{\left(R,G,B\right)max-\left(R,G,B\right)min}+0{}^{\circ},\;\;\;if\;\left(R,G,B\right)max=R,G\geq B\\ 60{}^{\circ}\cdot \frac{G-B}{\left(R,G,B\right)max-\left(R,G,B\right)min}+360{}^{\circ},if\;\left(R,G,B\right)max=R,G<B\\ 60{}^{\circ}\cdot \frac{B-R}{\left(R,G,B\right)max-\left(R,G,B\right)min}+120{}^{\circ},\;\;\;if\;\left(R,G,B\right)max=G\\ 60{}^{\circ}\cdot \frac{R-G}{\left(R,G,B\right)max-\left(R,G,B\right)min}+240{}^{\circ},\;\;\;if\;\left(R,G,B\right)max=B \end{array}\right.$$

(2) $$S=\left\{\begin{array}{c} 0,if\;\left(R,G,B\right)max=0\\ \frac{\left(R,G,B\right)max-\left(R,G,B\right)\min}{\left(R,G,B\right)max},otherwise \end{array}\right.$$

(3) $$V=\left(R,G,B\right)max$$

where $\left(R,G,B\right)max$ is the largest value of *R*, *G*, and *B* in each pixel; and $\;\left(R,G,B\right)min$ is the smallest value of *R*, *G*, and *B* in each pixel.

**Figure 3:**
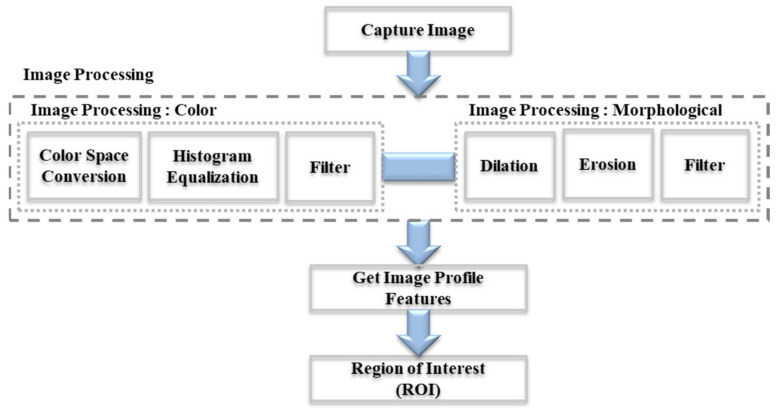
Flow chart of the image-processing module.

In order to achieve the primary purpose of object recognition and AUV tracking, the target characteristics captured by the cameras are implemented in the navigation after analysis through the use of AUV and recognition technology during underwater object recognition. The major challenge in using image-processing technology for detection is that objects appear differently underwater compared to in air. Consequently, in this study, some characteristics in the images were enhanced for the computer to process the information. A red ball was designated as the target because red is extremely observable in underwater environments. Since the HSV color space can be used to reduce the effects caused by changes in ambient light sources and to increase the resolution, the image-processing technology is still able to recognize the object correctly in a similarly colored background. In addition, the respective color attenuation is small with increasing distance. Therefore, some processing methods are adopted to eliminate the influence of noise (attenuation and scattering of light by suspended particles) and improve detection. The image-processing methods of this study include image capture, color space conversion, image binarization, filter, and morphology operation [48]. The advantage of these image-processing methods is to obtain both the color and morphological information of the object in an efficient way for further processing. The calculation procedure and the procedure for the proposed methods are shown in Figure 3. 

**Figure 4:**
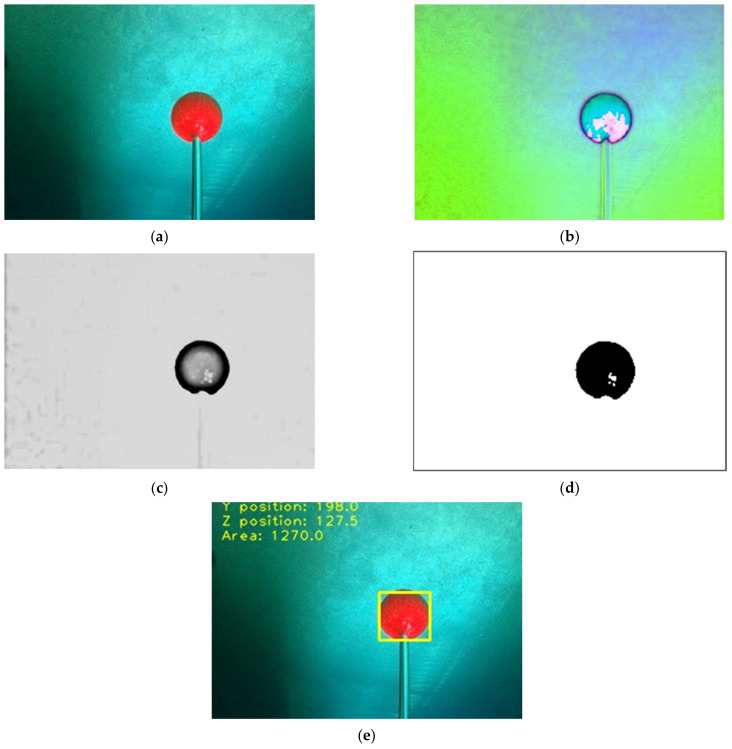
The procedure of image processing: (**a**) capturing the video from the AUV front camera; (**b**) conversion from the RGB color space to the HSV color space; (**c**) histogram equalization, binarization, and filter; (**d**) filter and morphology of dilation and erosion; and (**e**) obtaining the color features of the object.

The first step in the image recognition procedure is obtaining a continuous image from the image module in the fore part, as shown in Figure 4a. Subsequently, the image color space is converted from an RGB color space to an HSV color space, and the brightness dimension of the image is calculated as shown in Figure 4b. Next, the histogram equalization, image binarization, and image filter process are performed [49], as shown in Figure 4c. After the conversion from the RGB color space to the HSV color space is completed, the values of HSV can be designed as the threshold in the binarization according to the environmental change. When it is implemented in the real environment, the threshold values of HSV can be fine-tuned and designed to improve the recognition ability. Subsequently, the erosion and dilation basic methods are applied morphologically [50], as shown in Figure 4d.

As shown in Figure 4e, the features of the object could be identified using color recognition and selection of the region of interest (ROI) area. Specifically, the features are firstly detected by using the Canny edge detector [51], and then they are determined by adopting the Teh-Chin Algorithm [52] for the ROI processing of the target. By calculating the ROI area center of the object on the AUV image coordinate and the area of the selection, the rate between sizes of the object can be estimated [53], and then the AUV is able to complete the calculation process for real-time dynamic recognition of the image features. Eventually, the image coordinate and the ROI area can be defined as the object-tracking control parameters, i.e., the orientations and the distance [51].

**Figure 5:**
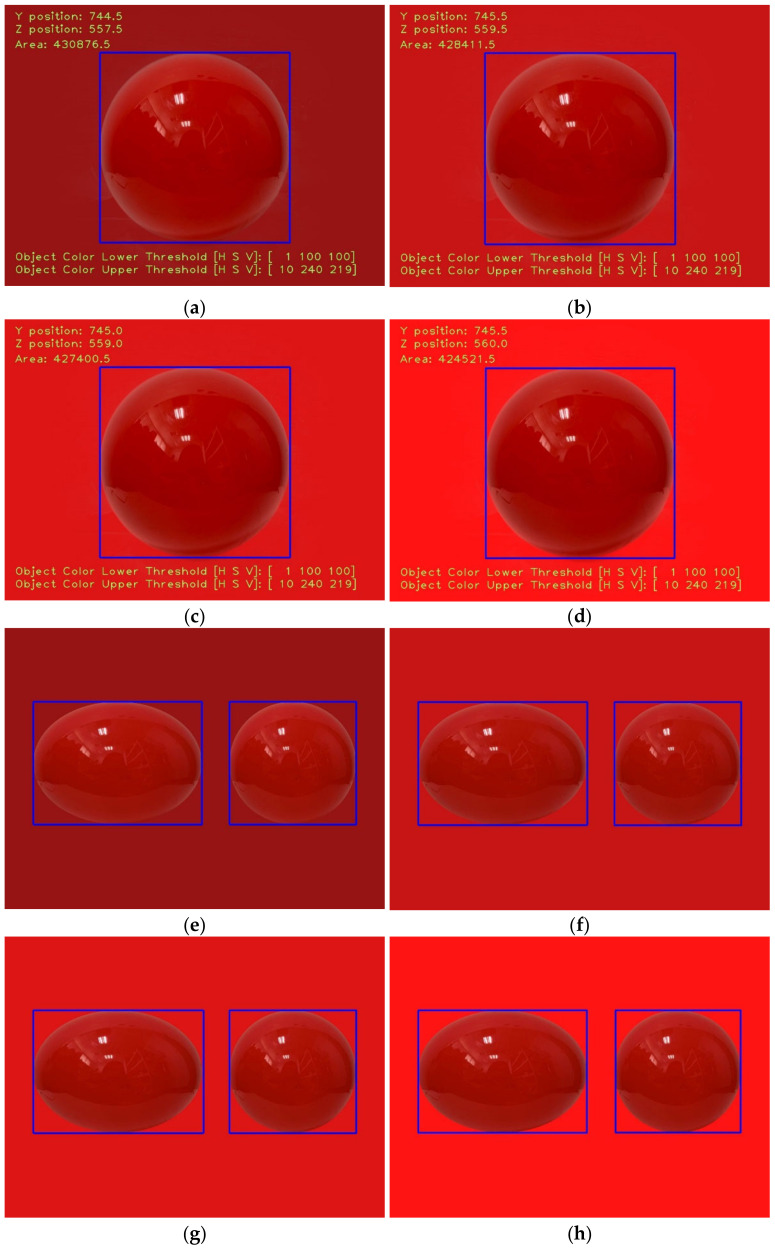
The ROI selections for (**a**) case A, (**b**) case B, (**c**) case C, (**d**) case D, (**e**) case E, (**f**) case F, (**g**) case G, and (**h**) case H, respectively.

In order to discuss the robustness of the present image-processing method, an experiment with similar objects and similarly colored backgrounds was implemented, as shown in Table 1 and Figure 5a–h. In the experiment, the thresholds of the HSV values when identifying the objects in similarly colored backgrounds were analyzed. It is apparent that the image-processing methods in this study are able to identify the object by adjusting the thresholds of the HSV values. However, if there are two different objects of the same color, e.g., a sphere and an ellipsoid, the image-processing methods would select both of them as the ROI simultaneously. In a future study, a more robust image-processing method, such as a Region-Based Convolutional Neural Network (R-CNN) [54], would be considered to recognize the features of the different morphologies in the same color.

## 4. Image-Based Navigation System

Since the underwater image-based navigation system is a combination of color recognition and fuzzy controllers, the details of the technologies would be introduced in this section.

### 4.1. Relationships between Different Coordinate Systems

**Figure 6:**
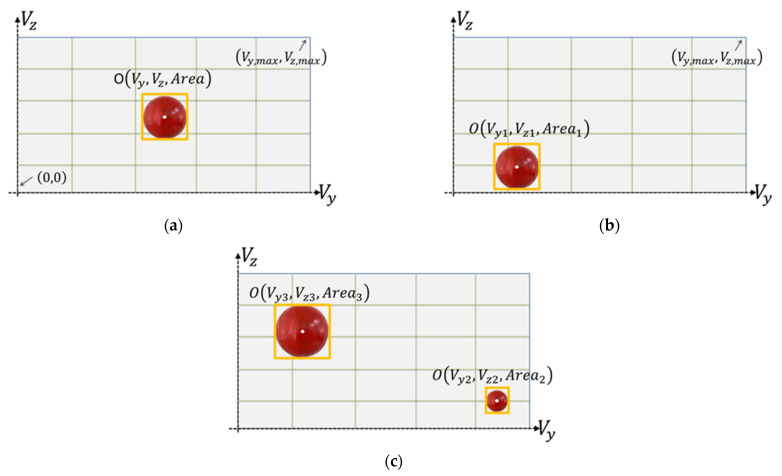
Definitions of the tracking control parameters values: (**a**) the benchmark, (**b**) the orientations, and (**c**) the distance.

The AUV control coordinate system is defined using the body-fixed coordinate system, and the trajectory relationships of the AUV in space are defined using the earth-fixed coordinate system. Figure 6 shows the vehicle image coordinate system with the red ball as an object. The coordinate value and area value $O\left(V_{y},V_{z},Area\right)$ of the object within the image coordinates were calculated to define the relative horizontal and vertical relationship between the AUV and the object and to define the relative distance between the AUV and the object.

Figure 6a shows the benchmark visual image when the relative distance of the target matches the requirements, at which the forward speed and the heading angle are set at zero. Compared to the visual image in Figure 6a, the visual image shown in Figure 6b can be defined as the operation strategy of turning left and down when the object is in the lower-left corner of the AUV. On the other hand, the visual image shown in Figure 6c can be identified as the operation strategy with backward speed when the area of the object is larger than that in Figure 6a.

**Figure 7:**
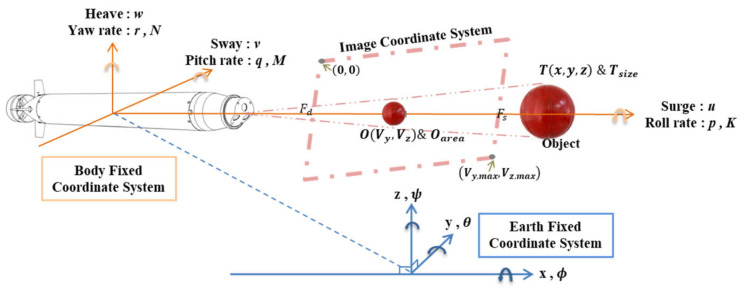
Relationship between the earth-fixed, body-fixed, and vehicle image coordinate systems.

Furthermore, the relative relationship and the distance between the target object and the AUV in space can be estimated, as shown in Figure 7. The earth-fixed coordinates of target object *T* are set as $T\left(x,y,z\right)$; the image target *O*, which is projected to and captured by the camera inside the AUV, is defined as $O\left(V_{y},V_{z}\right)$ in the image coordinate system; and the relationship is illustrated in Equation (4), with $O_{area}$ and $T_{size}$ (defined in Equation (5)) representing the area size of the target object in the earth-fixed coordinate system and vehicle image coordinate system, respectively, where $F_{d}$ is the focal length of the vehicle camera and $F_{s}$ is a fixed scale factor.

(4) $$T\left(x,y,z\right)\;\Rightarrow O\left(V_{y},V_{z}\right)=\left[\begin{array}{c} i\\ j \end{array}\right]=F_{d}/x\left[\begin{array}{c} y\\ z \end{array}\right]$$

(5) $$T_{size}=F_{s}\cdot O_{area}$$

### 4.2. Fuzzy Logic Control (FLC)

The navigation drive and propulsion control use fuzzy logic control (FLC) [55], processing nonstable signals with a simple mathematical model based on nonlinear control logic. When unstable signals cannot provide accurate values for performing the control calculations, a defined value range model with high tolerance is used as the constraint condition to form fuzzy controls. In the application of digital computer controls, the advantages of FLC advantages are its similarity to human thinking models and the ability to implement gentle processing protocols for unstable signal sources [56].

Fuzzy sets are the theoretical basis of FLC, and they are defined by the characteristic function as below:

(6) $$S_{A}\left(x\right)=\left\{\begin{array}{c} 1\;\;\;,\;\;if\;x\in \;A\\ 0\;\;\;,\;\;if\;x\notin \;A \end{array}\right.$$

where x is expressed as an element, and $S_{A}\left(x\right)$ is the characteristic function of *A*.

Zadeh [55] quantified and calculated fuzzy sets from the precise value of the traditional set through a predefined membership function. When membership function of fuzzy set *A* defined into $U_{A}\left(x\right)$ is discrete, then fuzzy set *A* can be expressed as Equation (7). If membership function $U_{A}\left(x\right)$ is continuous, then *A* is expressed as Equation (8).

(7) $$A=\frac{U_{A}\left(x_{1}\right)}{x_{1}}+\frac{U_{A}\left(x_{2}\right)}{x_{2}}+\ldots +\frac{U_{A}\left(x_{n}\right)}{x_{n}}$$

(8) $$A=\int_{0}^{\infty }\frac{U_{A}\left(x\right)}{x}$$

where U is an all fuzzy set.

In this study, fuzzy logic is applied on three groups of single-in, single-out controllers. Fuzzy inference uses generalized modus ponens [57] and establishes a fuzzy rule base by using the Tsukamoto fuzzy model through the IF-THEN inference rule, as shown in Equation (9) [58]. In the system, when the input value is imported into the fuzzy controller, the monotonic membership function corresponding to the fuzzy rule library is subjected to the defuzzifier and is mapped onto the value range to obtain the control coefficients, where $C_{1}$, $C_{2}$, and $C_{3}$ represent the monotonic membership functions.

(9) $$\begin{array}{c} If\;a\;is\;small,\;then\;b\;is\;C_{1}\\ If\;a\;is\;medium,\;then\;b\;is\;C_{2}\\ If\;a\;is\;large,\;then\;b\;is\;C_{3} \end{array}$$

The maneuverability of the AUV is a coupled, complex integrated system that is nonlinearly coupled, and numerous undefined coefficients are present in the differential equation of its six degrees of motion. The advantages of FLC in AUV applications, as highlighted by Smith, et al. [59], are integrated into the motion drive and propulsion control system to form the fuzzy control framework of the yaw, pitch angle, and the propeller revolution speed.

### 4.3. Servomotor Controller Design

To enable stable horizontal control of the AUV at depths, its ballast arrangement is in a state of neutral buoyancy deviated toward positive buoyancy (0.8 kgf). Therefore, only the control of the vertical rudder plane angle ($\delta_{V}$) is discussed. To maintain the target at the image centre, the target detected by the camera is calculated by the controller. The centre is defined at 0° of the servomotor, and every graduation is 5°. The maximum angle change is ±30°. When the centroid of the object is on the right side of the image, the heading control is at a negative value.

The control principle of this system dominated the servomotor. When the target is on the right side of the image, the centre distance is subtracted from the coordinate as the error value of the FLC. The area where the target object is displayed on the vehicle camera image coordinate $\;O\left(V_{y}\right)$ is the FLC input variable for defining the following six fuzzy values: strongleft, left, middle, right, strongright, and Vright.

**Figure 8:**
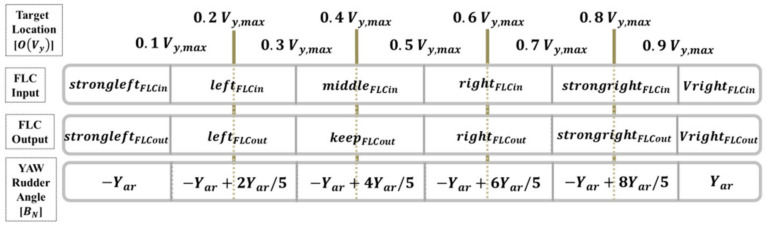
Relationship between the target’s image horizontal coordinate axis and the FLC yaw angle.

The FLC calculation and control target is the AUV yaw angle $\left(B_{N}\right)$, ranging from −30° to 30°; the six defined output rudder controls are strongleft, left, middle, right, strongright, and Vright. The FLC framework of the yaw angle is presented in Figure 8, Equation (10), and Equation (11), respectively.

(10) $$\begin{array}{c} If\;O\left(V_{y}\right)<(0.1V_{y,max})\;is\;strongleft_{FLCin}\\ \;\;\;then\;strongleft_{FLCout}\;is-Y_{ar}\\ If\;(0.1V_{y,max})\leq O\left(V_{y}\right)<(0.3V_{y,max})\;is\;left_{FLCin}\\ \;\;\;then\;left_{FLCout}\;is-Y_{ar}+2Y_{ar}/5\\ If\;(0.3V_{y,max})\leq O\left(V_{y}\right)<(0.5V_{y,max})\;is\;middle_{FLCin}\;\\ \;\;\;then\;keep_{FLCout}\;is-Y_{ar}+4Y_{ar}/5\\ If\;(0.5V_{y,max})\leq O\left(V_{y}\right)<(0.7V_{y,max})\;is\;right_{FLCin}\\ \;\;\;then\;right_{FLCout}\;is-Y_{ar}+6Y_{ar}/5\\ If\;(0.7V_{y,max})\leq O\left(V_{y}\right)<(0.9V_{y,max})\;is\;strongright_{FLCin}\\ \;\;\;then\;strongright_{FLCout}\;is-Y_{ar}+8Y_{ar}/5\\ If\;(0.9V_{y,max})\leq O\left(V_{y}\right)\;is\;Vright_{FLCin}\\ \;\;\;then\;Vright_{FLCout}\;is\;Y_{ar} \end{array}$$

(11) $$B_{N}=\left\{\begin{array}{c} -Y_{ar}\;,\;if\;O\left(V_{y}\right)<(0.1V_{y,max}{)}_{\;}\\ -Y_{ar}+2Y_{ar}/5\;,\;if\;(0.1V_{y,max})\leq O\left(V_{y}\right)<(0.3V_{y,max}{)}_{\;}\\ -Y_{ar}+4Y_{ar}/5\;,\;if\;(0.3V_{y,max})\leq O\left(V_{y}\right)<(0.5V_{y,max}{)}_{\;}\\ -Y_{ar}+6Y_{ar}/5\;,\;if\;(0.5V_{y,max})\leq O\left(V_{y}\right)<(0.7V_{y,max}{)}_{\;}\\ -Y_{ar}+8Y_{ar}/5\;,\;if\;(0.7V_{y,max})\leq O\left(V_{y}\right)<(0.9V_{y,max}{)}_{\;}\\ Y_{ar}\;,\;if\;(0.9V_{y,max})\leq O{\left(V_{y}\right)}_{\;} \end{array}\right.$$

where $\pm \;Y_{ar}$ is the maximum range value that the AUV vertical rudders are able to control.

**Figure 9:**
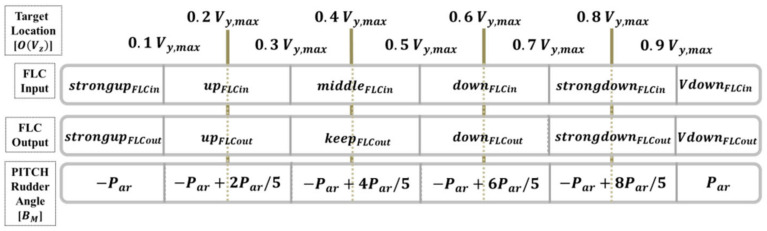
Relationship between the target’s image vertical coordinate axis and the FLC pitch angle.

Regarding the vehicle’s pitch angle, on the basis of the image feedback framework and by using the fuzzy logical controller FLC, the input variable calculates and controls the AUV pitch angle $\left(B_{M}\right)\;$ according to the image coordinates of the target object on the image vertical coordinate $T\left(V_{z}\right)$. Six input fuzzy values are defined: strongup, up, middle, down, strongdown, and Vdown. The vehicle’s pitch angle is the calculation and control target, ranging from −30° to positive 30°; the six defined fuzzy values are strongup, up, middle, down, strongdown, and Vdown. The pitch angle FLC framework is illustrated in Figure 9, and expressed in Equation (12).

(12) $$B_{M}=\left\{\begin{array}{c} -P_{ar}\;,\;if\;O\left(V_{z}\right)<(0.1V_{z,max}{)}_{\;}\\ -P_{ar}+2P_{ar}/5\;,\;if\;(0.1V_{z,max})\leq O\left(V_{z}\right)<(0.3V_{z,max})\;{\;}_{\;}\\ -P_{ar}+4P_{ar}/5\;,\;if\;(0.3V_{z,max})\leq O\left(V_{z}\right)<(0.5V_{z,max}{)}_{\;}\\ -P_{ar}+6P_{ar}/5\;,\;if\;(0.5V_{z,max})\leq O\left(V_{z}\right)<(0.7V_{z,max}){\;}_{\;}\\ -P_{ar}+8P_{ar}/5\;,\;if\;(0.7V_{z,max})\leq O\left(V_{z}\right)<(0.9V_{z,max}{)}_{\;}\\ P_{ar}\;,\;if\;(0.9V_{z,max})\leq O{\left(V_{z}\right)}_{\;} \end{array}\right.$$

where $\pm \;P_{ar}$ is the maximum range value that the AUV horizontal rudders are able to control.

### 4.4. Propeller Controller Design

The control method of the AUV’s propeller revolution speed depends on the area of the target. After the target is captured in the image, the area is calculated to define the revolution speed configuration. The judgment method is that the larger the area is, the lower the revolution speed is. By contrast, the smaller the area is, the higher the revolution speed is.

**Figure 10:**
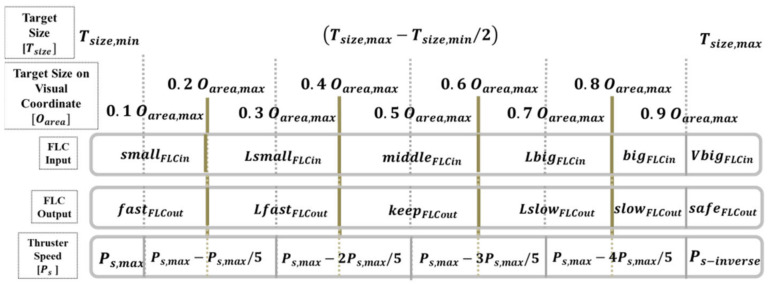
Relationship between the target’s size within the image coordinates and the FLC propeller revolution speed.

The FLC input variable calculates and controls the AUV propeller revolution speed $\left(P_{s}\right)$ according to the target object coordinate $T\left(V_{z}\right)$ and size $T_{size}$ displayed on the image coordinate. Six input fuzzy values are defined: small, Lsmall, middle, Lbig, big, and Vbig. The propeller revolution speed $\left(P_{s}\right)$ is the output variable, with a range between $P_{s-inverse}\;$ and $P_{s,max}$; the six defined fuzzy values are fast, Lfast, keep, Lslow, slow, and safe. The FLC framework of the propeller revolution speed is presented in Figure 10 and can be expressed in Equation (13).

(13) $$P_{s}=\left\{\begin{array}{c} P_{s,max}\;,if\;O_{area}<{\left(0.1O_{area,max}\right)}_{\;}\\ or\;O\left(V_{z}\right)<(0.1V_{z,max}{)}_{\;}\\ P_{s-inverse}\;,if\;O_{size}\geq {\left(0.9O_{area,max}\right)}_{\;}\\ \;or\;(0.1V_{z,max})\leq O\left(V_{z}\right)<(0.3V_{z,max}{)}_{\;}\\ P_{s,max}-P_{s,max}/5\;{,}_{\;}\\ if\left(0.1O_{area,max}\right)\leq O_{area}<{\left(0.3O_{area,max}\right)}_{\;}\\ or\;(0.3V_{z,max})\leq O\left(V_{z}\right)<(0.5V_{z,max}\;{)}_{\;}\\ P_{s,max}-2P_{s,max}/5\;{,}_{\;}\\ if\;\left(0.3O_{area,max}\right)\leq O_{area}<{\left(0.5O_{area,max}\right)}_{\;}\\ or\;(0.5V_{z,max})\leq O\left(V_{z}\right)<(0.7V_{z,max}{)}_{\;}\\ P_{s,max}-3P_{s,max}/5\;{,}_{\;}\\ if\;\left(0.5O_{area,max}\right)\leq O_{area}<{\left(0.7O_{area,max}\right)}_{\;}\\ or\;(0.7V_{z,max})\leq O\left(V_{z}\right)<(0.9V_{z,max}{)}_{\;}\\ P_{s,max}-4P_{s,max}/5\;{,}_{\;}\\ if\;\left(0.7O_{area,max}\right)\leq O_{area}<\left(0.9O_{area,max}\right){\;}_{\;}\\ or\;(0.9V_{z,max})\leq O{\left(V_{z}\right)}_{\;} \end{array}\right.$$

where $P_{s,max}$ is the maximum propeller revolution speed of the AUV in the towing tank experiment, and $P_{s-inverse}\;$ is the reverse propeller revolution speed when the safety distance control mechanism is activated.

### 4.5. Extended Kalman Filter (EKF)

KFs [60] are based on linear assumptions and are estimated based on the state estimation value of the previous moment and the state observation value of the current moment. Because noise or unstable measurement values need not be eliminated and processed in advance, estimations can be performed with recursive data processing algorithms in the time domain. Furthermore, observation data and historical data need not be logged. However, through each regression, the predicted value and the observed value are weighted to obtain an estimate close to the dynamic target.

Because the KF is an estimate based on linear assumptions, divergence exists when it is used on nonlinear systems. Therefore, many studies have expanded the application of KFs to nonlinear systems, and the core processing protocol involves the linearization of nonlinear systems. Sorenson and Stubberud [61] proposed the EKF, and Alspach and Sorenson [62] proposed Bayesian estimation, which linearizes nonlinear systems by using a Gaussian distribution approximation. Regarding other methods for linearizing nonlinear systems, Chen [63] argued that an expanded Taylor series can be used to approximate nonlinear systems, and the nonlinear system can be transformed into a linearized system by simplifying or ignoring the higher order terms of the nonlinear system. Although the EKF uses approximation methods to solve nonlinear system problems, it conversely lowers the precision of the control model and drastically increases the amount of data processing. Furthermore, when highly variable coefficients are present in the nonlinear system, the estimation results diverge and result in estimation control failure.

**Figure 11:**
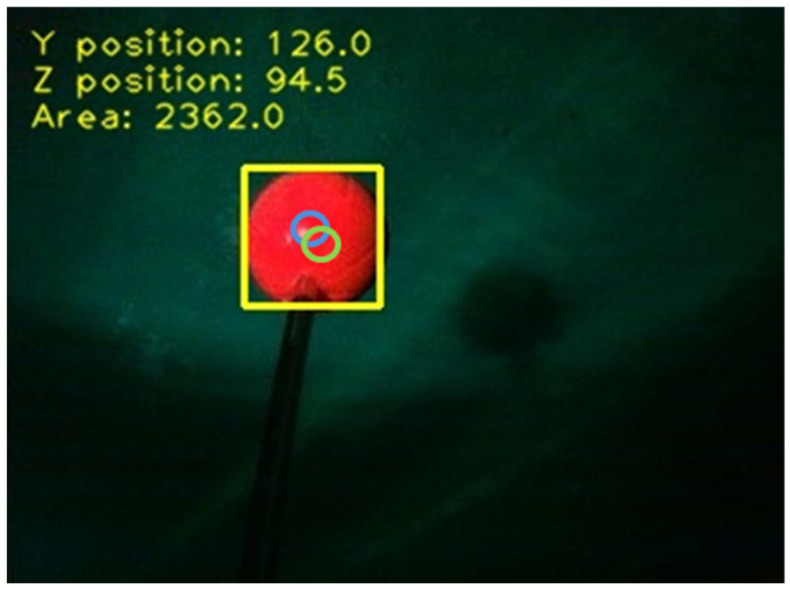
The recognition results of the image with the EKF.

Since the integration of the camera measurement and the controller operation inevitably causes signal delays, the system performance would be improved by using the EKF. In this study, the purpose of the dynamic guidance experiment is to understand both the qualitative and quantitative results of the system performance. Therefore, the red ball is selected as the target in the experiment due to its size and geometry. The free movements of the red ball are tracked with the control of the propeller to ensure that the target remained in the frame. The coordinates of the target are read to estimate its next coordinates. Figure 11 shows the underwater image-recognition results. The coordinates are documented at a speed of 15 frames per second, and the largest area in the image is identified and framed with a rectangle. The centre of the rectangle represents the position of the object. The blue circle is the coordinate of the actual object, and the green circle is the coordinate predicted by the EKF. Differences are observed between the actual and predicted positions.

## 5. Experimental Results and Discussion

The experiments were conducted in the stability water tank and towing tank at Department of Systems and Naval Mechatronic Engineering of National Cheng Kung University (NCKU). The underwater image recognition tests are conducted primarily in the stability water tank. The experiments of the FLC system were carried out in the towing tank, and the comparison results for the EKF were added for verification. In this study, underwater image processing was conducted in the stability water tank by using a dual-lens camera with varying brightness values. The area and coordinate data were calculated by processing the image information in various conditions. The recognition results were matched with an FLC and EKF to be included in the AUV control system. The underwater object tracking of the AUV was adjusted dynamically with the image-recognition results.

### 5.1. Experimental Environment and Equipment

**Figure 12:**
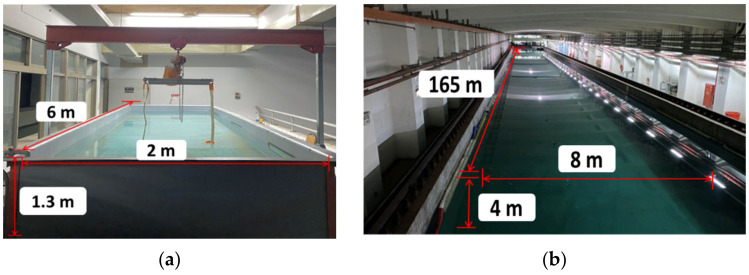
The exterior and the dimension of (**a**) the stability water tank, and (**b**) the towing tank.

The stability water tank of NCKU is 6 m in length, 2 m in width, and 1.3 m in depth. The towing tank of NCKU is 165 m in length and 8 m in width, and the working water depth is 4 m. The exterior and the dimensions of the stability water tank and the towing tank are indicated in Figure 12a,b, respectively.

### 5.2. Experimental Procedure

**Figure 13:**
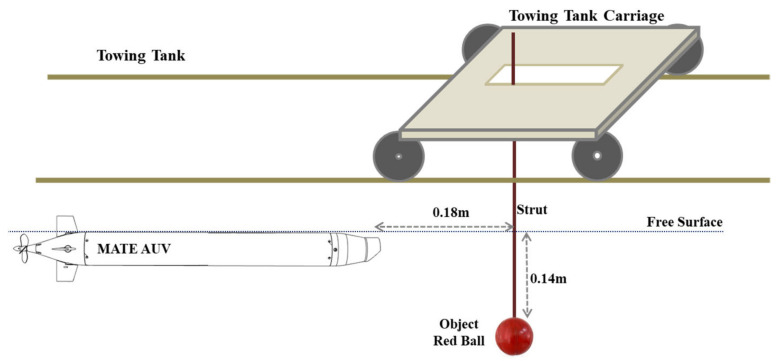
Schematic of the AUV and target object in the dynamic experiment.

**Figure 14:**
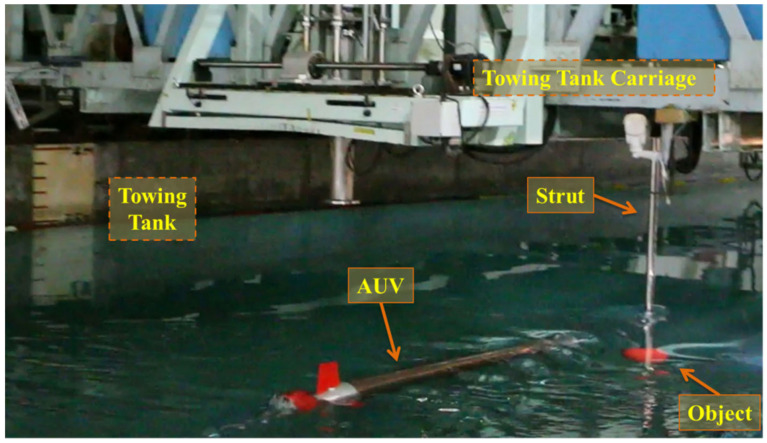
Environment of the dynamic experiment.

This study was conducted in both a stability water tank and a towing tank. The stability water tank was used to test the vehicle’s attitude adjustment, buoyancy balance, water-tightness, payload control, and examined for image-recognition processing. Finally, the test results were introduced into the AUV for the dynamic experiment in the towing tank. The target used for the underwater recognition was a red ball of 24 cm in diameter, fastened to the support frame, and installed securely onto the towing carriage. The object was submerged in the water; the top of the object was 0.14 m away from the water surface, and the minimal safety distance between the object and the AUV was 0.18 m; the experimental environment is presented in Figure 13 and Figure 14. During the experiment, the movement of the towing carriage drove the object, and the image-based navigation system was tested under various speed conditions of the object.

**Figure 15:**
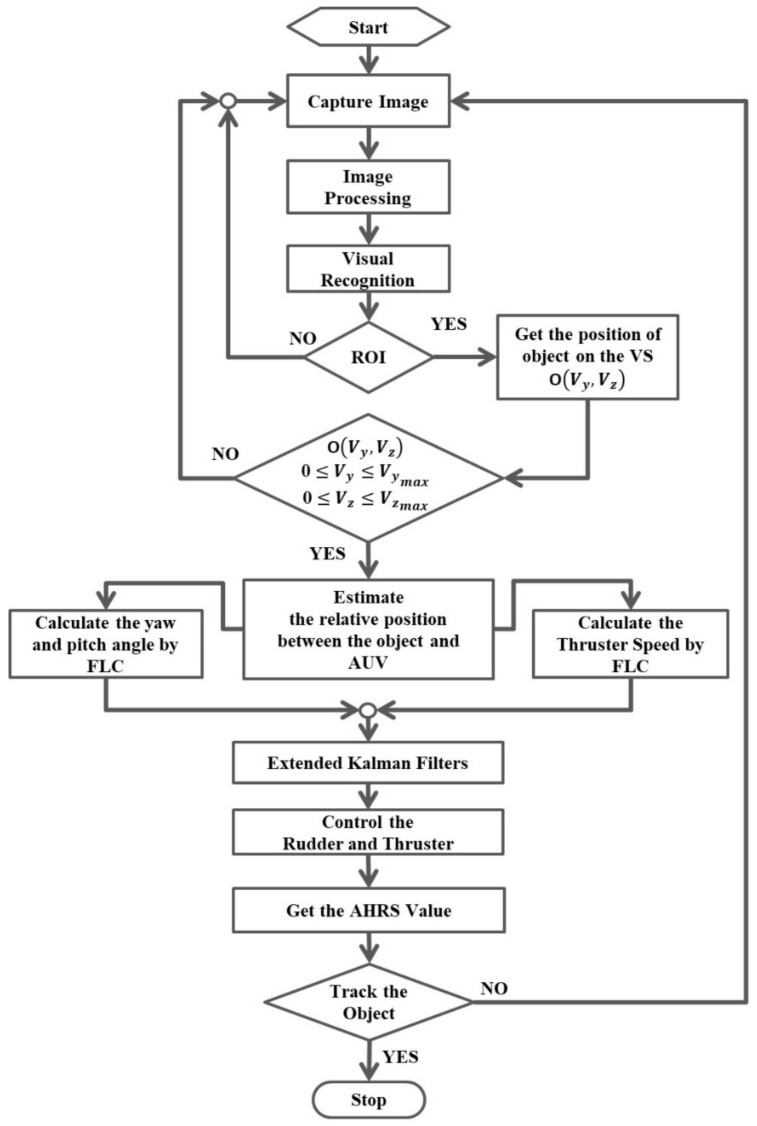
The flow chart of the image-based navigation system in the AUV.

During the tests in the towing tank, to eliminate the noise generated in the measurement process and achieve accurate estimation results at the current position, the influence of the EKF on the predicted coordinate results is further discussed in this study. The flow chart of the image-based navigation system is introduced in Figure 15.

In the flowchart, the object mapped onto the image coordinate system $O\left(V_{y},V_{z}\right)$ can be calculated when the ROI is selected. Subsequently, the information obtained from the ROI would enter the procedure for the propeller and rudder controls by using the FLC. In order to improve performances of the propeller and rudder controls, the output control values, i.e., yaw angle $\left(B_{N}\right)$, pitch angle $\left(B_{M}\right)$, and propeller revolution speed $\left(P_{s}\right)$ of the FLC, in the preceding 15 s would be adopted as the input data of the EKF, and the AUV motion control can be performed by using the predicted data of the EKF.

### 5.3. Experimental Results and Data Analysis

**Figure 16:**
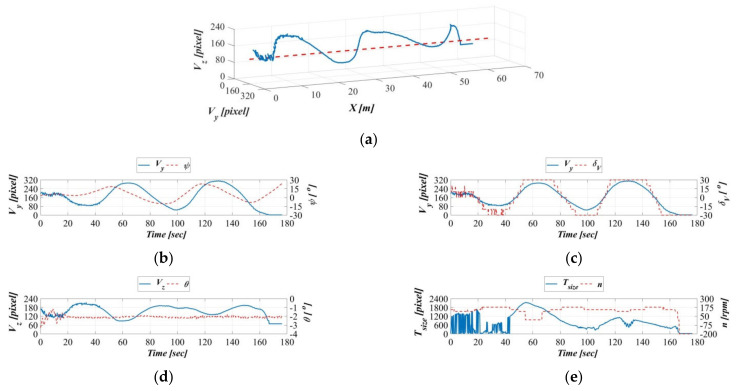
Dynamic guidance experiment with a towing speed of 0.2 m/s. (**a**) Displacement trajectory on the image coordinates, (**b**) time series of the image coordinate Y−axis and AUV yaw angle, (**c**) time series of image coordinate Y−axis and AUV vertical rudder, (**d**) time series of the image coordinate Z−axis and the AUV vertical rudder, and (**e**) time series of the image coordinate surface area and the AUV propeller revolution speed.

**Figure 17:**
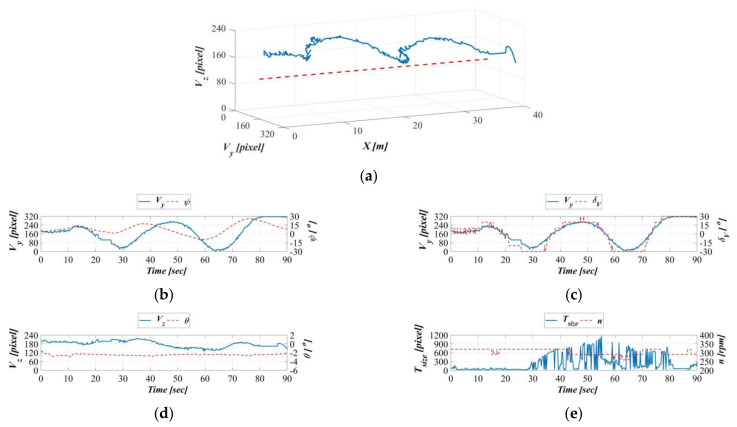
Dynamic guidance experiment with a towing speed of 0.4 m/s. (**a**) Displacement trajectory on the image coordinates, (**b**) time series of the image coordinate Y−axis and AUV yaw angle, (**c**) time series of image coordinate Y−axis and AUV vertical rudder, (**d**) time series of the image coordinate Z−axis and the AUV vertical rudder, and (**e**) time series of the image coordinate surface area and the AUV propeller revolution speed.

**Figure 18:**
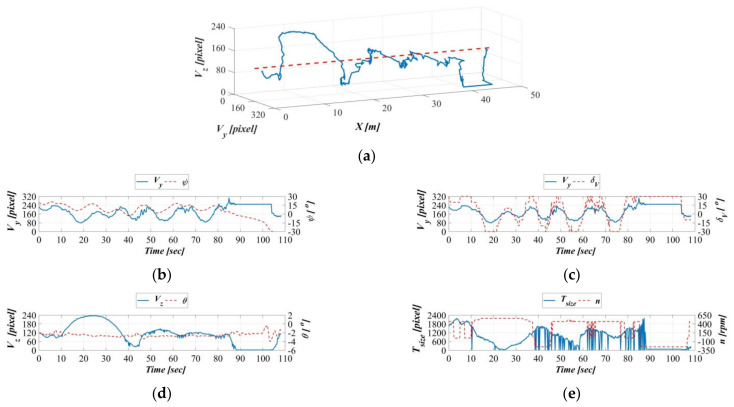
Dynamic guidance experiment with a towing speed of 0.6 m/s. (**a**) Displacement trajectory on the image coordinates, (**b**) time series of the image coordinate Y−axis and AUV yaw angle, (**c**) time series of image coordinate Y−axis and AUV vertical rudder, (**d**) time series of the image coordinate Z−axis and the AUV vertical rudder, and (**e**) time series of the image coordinate surface area and the AUV propeller revolution speed.

**Figure 19:**
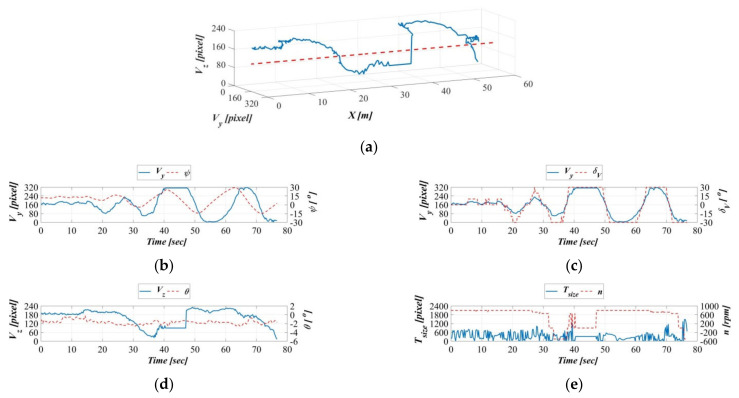
Dynamic guidance experiment with a towing speed of 0.8 m/s. (**a**) Displacement trajectory on the image coordinates, (**b**) time series of the image coordinate Y−axis and AUV yaw angle, (**c**) time series of image coordinate Y−axis and AUV vertical rudder, (**d**) time series of the image coordinate Z−axis and the AUV vertical rudder, and (**e**) time series of the image coordinate surface area and the AUV propeller revolution speed.

Figure 16, Figure 17, Figure 18 and Figure 19 present the AHRS measurements when the object moved at speeds of 0.2, 0.4, 0.6, and 0.8 m/s during the dynamic experiments. Figure 16a–e presents the results of the dynamic experiment with the object moving at 0.2 m/s; in order, the figure presents the relationship of the object’s displacement trajectory in the vehicle’s image coordinate system with that in the earth-fixed coordinates system (Figure 16a), the time series of the target object on the vehicle’s image coordinate system Y-axis and the AUV’s yaw angle on the body-fixed coordinates system (Figure 16b), the time series of the target object on the vehicle image coordinate system Y-axis and the AUV vertical rudder (Figure 16c), the time series of the target object on the vehicle image coordinate system Z-axis and the AUV’s pitch angle on the body-fixed coordinates system (Figure 16d), and the time series of the target’s area size on the vehicle image coordinate system and the AUV propeller revolution speed (Figure 16e).

The results in Figure 16a indicate that, except for the displacement trajectory exhibiting greater fluctuations as a result of the towing carriage decelerating in the final stage, the target object is controlled in the area range defined by the image coordinate $O\left(V_{y},V_{z}\right)$. Figure 16b demonstrates consistent trends between the object on the vehicle image coordinate system Y-axis and the AUV yaw angle on the body-fixed coordinate system; this indicates that the image-based navigation system can track the target object’s horizontal movement changes in real time. Figure 16c reveals that the target on the vehicle image coordinate system Y-axis and the AUV vertical rudder are almost synchronized, indicating that the motion controller can respond to changes in the vehicle image coordinate system Y-axis in real time. Figure 16d demonstrates consistent trends between the object on the vehicle image coordinate system Z-axis and the AUV pitch angle on the body-fixed coordinate system; this indicates that the image-based navigation system can track the object’s vertical movement changes in real time. Figure 16e demonstrates exceedingly similar trends between the AUV propeller revolution speed and changes in the area size of the target object, indicating that the AUV propeller revolution speed can reflect surface area changes in the vehicle image coordinate system in real time.

Because of the acceleration of the object’s moving speed, as illustrated in Figure 17a, Figure 18a, and Figure 19a, the object exhibited greater fluctuations in displacement, but these are still controlled within the area range defined by the image coordinate $O\left(V_{y},V_{z}\right)$. Figure 17b, Figure 18b, and Figure 19b indicate gradual increasing changes to the object on the vehicle image coordinate system Y-axis and the AUV yaw angle on the body-fixed coordinate system, compared to Figure 16b. Similarly, the time series of vertical rudders in Figure 17c, Figure 18c, and Figure 19c exhibit consistency with the changes along the vehicle image coordinate system Y-axis. Furthermore, with the acceleration of the towing carriage, the change fluctuations in the target object along the vehicle image coordinate system Z-axis and the AUV’s pitch angles in the body-fixed coordinate system also increased (Figure 17d, Figure 18d, and Figure 19d). A comparison of Figure 16e, Figure 17e, Figure 18e, and Figure 19e demonstrates little difference in propeller revolution speed; this is because the moving speed of the target objects is relatively stable, and as a result, surface area changes in the vehicle image coordinate system are also more stable.

**Figure 20:**
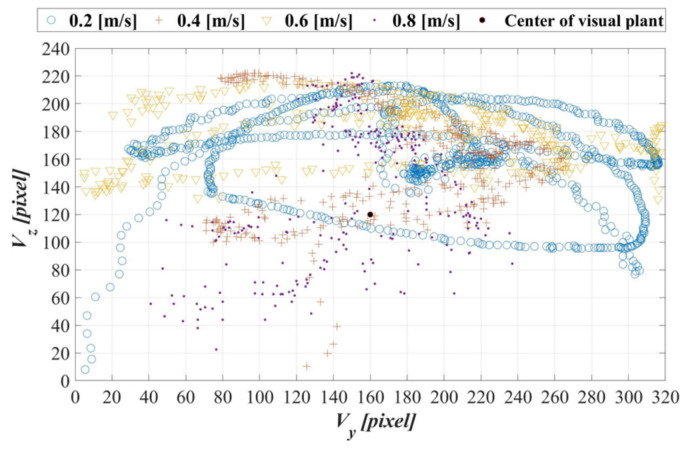
Image coordinate system logs of the AUV in dynamic guidance experiments with towing speeds of 0.2, 0.4, 0.6, and 0.8 m/s.

Figure 20 presents the image coordinate system logs of the AUV in the dynamic guidance experiments with towing speeds of 0.2, 0.4, 0.6, and 0.8 m/s. The experimental results indicated that, under these four towing speeds, the AUV could track the object in real-time and maintain the target object in the vehicle image coordinate system. With the acceleration of the towing speed, the experimental data gradually became more centralized, especially in the image coordinate Y-axis ($V_{y}$). This finding highlights that the moving speed of the target object is a factor affecting the AUV tracking efficiency.

**Figure 21:**
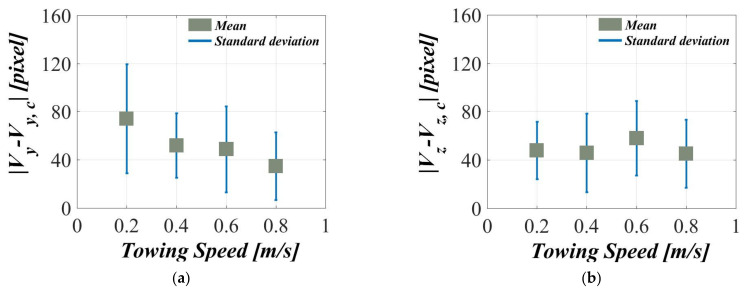
Statistical results of the dynamic guidance experiments in different towing speeds: the mean values and standard deviations of (**a**) $\left|V_{y}-V_{y,\;c}\right|$ and (**b**) $\left|V_{z}-V_{z,\;c}\right|$.

Figure 21a,b present the statistical results of $\left|V_{y}-V_{y,\;c}\right|$ and $\left|V_{z}-V_{z,\;c}\right|$ in the dynamic guidance experiments with towing speeds of 0.2, 0.4, 0.6, and 0.8 m/s. It is found in Figure 21a that the standard deviations of $\left|V_{y}-V_{y,\;c}\right|$ are 45.35, 26.83, 20.67, and 28.18 pixels. Furthermore, the standard deviations of $\left|V_{z}-V_{z,\;c}\right|$ in Figure 21b are 25.09, 32.54, 39.89, and 28.11 pixels. Meanwhile, the results indicate that the mean values of $\left|V_{y}-V_{y,\;c}\right|$ are 74.33, 51.97, 37.96, and 34.84 pixels, whereas the ones of $\left|V_{z}-V_{z,\;c}\right|$ are 43.97, 45.99, 44.21, and 45.33 pixels. As the towing speed accelerates, the experimental data of $\left|V_{y}-V_{y,\;c}\right|$ gradually become more centralized. However, the increase in the towing speed has less influence on the results of $\left|V_{z}-V_{z,\;c}\right|$. This is because the AUV, when it tracks the target object, can more easily maintain the target object at a safe distance at low speeds than at high speeds. However, this also slows down the real-time calibration of the yaw angle. The experimental results also reveal that the moving speed of the target object has little effect on the mean value of $\left|V_{z}-V_{z,\;c}\right|$ and standard deviation; instead, the moving speed has a closer relationship with the changes in the target object’s size, and as a result, effects on the real-time calibration of the pitch angle are minuscule when the moving speed is constant.

## 6. Conclusions

This study presents a technique for image recognition and a vehicle motion control method based on an image-based navigation system and implements it in an AUV of independent development. With the combination of the image-processing methods and the effective motion control method, a dynamic guidance task of low power consumption and high efficiency for the AUV can be achieved. The data of the body-fixed coordinates are mapped using the image coordinates to define the correspondence of the distance and the orientation between the target and the AUV. The features of the object could be identified using color recognition and selection of the ROI area. By calculating the ROI area center of the object on the AUV image coordinates and the area of the selection, the relative relationship and distance between the object and the AUV can be estimated. Furthermore, the attitude and the propeller revolution speed of the AUV can be adjusted automatically through establishing the FLC rule base. It is verified that the FLC control of the AUV can be performed well by using the predicted data of the EKF. It is realized in the experiment that real-time recognition is controlled to achieve operational performance at a towing speed up to 0.8 m/s. Finally, autonomous dynamic control on the rudders and the propeller has been successfully verified by testing the maneuverability of the AUV in the towing tank. Compared with our previous study [48], the advantage of this study is that the features of the object could be identified using color recognition and selection of the region of interest (ROI) area. Meanwhile, the FLC control of the AUV can be performed well by using the predicted data of the EKF.

## Figures and Tables

**Table 1:** The RGB values of the background colors and the HSV threshold values of the objects.

Test	Object	${\left(R,G,B\right)}_{background}$	${\left(H,S,V\right)}_{lower}$	${\left(H,S,V\right)}_{upper}$
A	Sphere	(150, 20, 20)	(1, 100, 100)	(10, 240, 219)
B		(200, 20, 20)	(1, 100, 100)	(10, 240, 219)
C		(220, 20, 20)	(1, 100, 100)	(10, 240, 219)
D		(255, 20, 20)	(1, 100, 100)	(10, 240, 219)
E	Sphere and Ellipsoid	(150, 20, 20)	(1, 100, 100)	(10, 240, 219)
F		(200, 20, 20)	(1, 100, 100)	(10, 240, 219)
G		(220, 20, 20)	(1, 100, 100)	(10, 240, 219)
H		(255, 20, 20)	(1, 100, 100)	(10, 240, 219)

## Data Availability

Data sharing not applicable.
